# Automatic Enrollment in Patient Portal Systems Mitigates the Digital Divide in Healthcare: An Interrupted Time Series Analysis of an Autoenrollment Workflow Intervention

**DOI:** 10.1007/s10916-024-02114-7

**Published:** 2024-10-08

**Authors:** Leila Milanfar, William Daniel Soulsby, Nicole Ling, Julie S. O’Brien, Aris Oates, Charles E. McCulloch

**Affiliations:** 1 School of Medicine, University of California, San Francisco, 505 Parnassus Ave, San Francisco, CA 94143 United States of America; 2 Department of Pediatrics, Division of Pediatric Rheumatology, University of California, San Francisco, San Francisco, CA United States of America; 3 Department of Pediatrics, Division of General Pediatrics, University of California, San Francisco, San Francisco, CA United States of America; 4 Department of Pediatrics, Division of Pediatric Nephrology, University of California, San Francisco, San Francisco, CA United States of America; 5 Department of Epidemiology and Biostatistics, University of California, San Francisco, San Francisco, CA United States of America

**Keywords:** Electronic health records, Healthcare disparities, Race factors, Health services accessibility, Health records, Personal

## Abstract

**Purpose:**

Racial and ethnic healthcare disparities require innovative solutions. Patient portals enable online access to health records and clinician communication and are associated with improved health outcomes. Nevertheless, a digital divide in access to such portals persist, especially among people of minoritized race and non-English-speakers. This study assesses the impact of automatic enrollment (autoenrollment) on patient portal activation rates among adult patients at the University of California, San Francisco (UCSF), with a focus on disparities by race, ethnicity, and primary language.

**Materials and methods:**

Starting March 2020, autoenrollment offers for patient portals were sent to UCSF adult patients aged 18 or older via text message. Analysis considered patient portal activation before and after the intervention, examining variations by race, ethnicity, and primary language. Descriptive statistics and an interrupted time series analysis were used to assess the intervention’s impact.

**Results:**

Autoenrollment increased patient portal activation rates among all adult patients and patients of minoritized races saw greater increases in activation rates than White patients. While initially not statistically significant, by the end of the surveillance period, we observed statistically significant increases in activation rates in Latinx (3.5-fold, p = < 0.001), Black (3.2-fold, *p* = 0.003), and Asian (3.1-fold, *p* = 0.002) patient populations when compared with White patients. Increased activation rates over time in patients with a preferred language other than English (13-fold) were also statistically significant (p = < 0.001) when compared with the increase in English preferred language patients.

**Conclusion:**

An organization-based workflow intervention that provided autoenrollment in patient portals via text message was associated with statistically significant mitigation of racial, ethnic, and language-based disparities in patient portal activation rates. Although promising, the autoenrollment intervention did not eliminate disparities in portal enrollment. More work must be done to close the digital divide in access to healthcare technology.

**Supplementary Information:**

The online version contains supplementary material available at 10.1007/s10916-024-02114-7.

## Introduction

The 2009 Health Information Technology for Economic and Clinical Health (HITECH) Act incentivized the meaningful use of healthcare information technology (HIT), leading to the widespread adoption of patient portals [1–4]. These online applications allow patients to access their electronic health records (EHR), view test results, refill medications, and communicate with their care team, resulting in improved patient outcomes and greater participation in preventative health measures5–18. However, disparities in access to and use of patient portals persist [19–35]. People from minoritized groups, including Black and Latinx people as well as people of lower socioeconomic status, have lower rates of portal access and use; these groups also face structural inequities and poorer health outcomes than their White or wealthy counterparts [19–46]. The inequitable allocation of HIT has resulted in a digital divide that threatens to further exacerbate health disparities [47–53].

Interventions to address this digital divide have focused on technical training, improvements in user experience, and providing internet-connected devices [27, 54–57]. Organization-based solutions have included improving system workflows related to awareness and enrollment in portals [27]. For instance, ensuring that all patients receive an offer of portal access codes can help mitigate disparities [58–62]. However, these modifications can increase staff workload and place the burden of activation on patients.

This study aimed to streamline the enrollment process through automation, drawing on models from other sectors, such as automatic voter registration [63–69]. At UCSF, all adult patients not yet enrolled in the patient portal received an auto-enrollment link via text message after their visit. The intervention aimed to increase overall portal enrollment and reduce racial, ethnic, and primary language disparities in portal uptake. By tracking enrollment trends, the study hypothesized that autoenrollment would improve portal access for all patients, particularly those from minoritized groups.

## Methods

### Study Overview

Beginning March 17, 2020, we initiated an automated, standard patient portal autoenrollment text message intervention for all patients 18 years of age or older seen at the University of California, San Francisco (UCSF) for an outpatient encounter who were not yet enrolled in the patient portal. Before, during, and throughout the intervention period, we analyzed differences between the immediate and sustained changes in enrollment trends by race, ethnicity, and primary language. Autoenrollment offers were not provided to pediatric patients given guardian verification requirements and privacy concerns among the adolescent population. However, general enrollment trends in the pediatric patient population were also tracked during this same period.

### Intervention

**Fig. 1:**
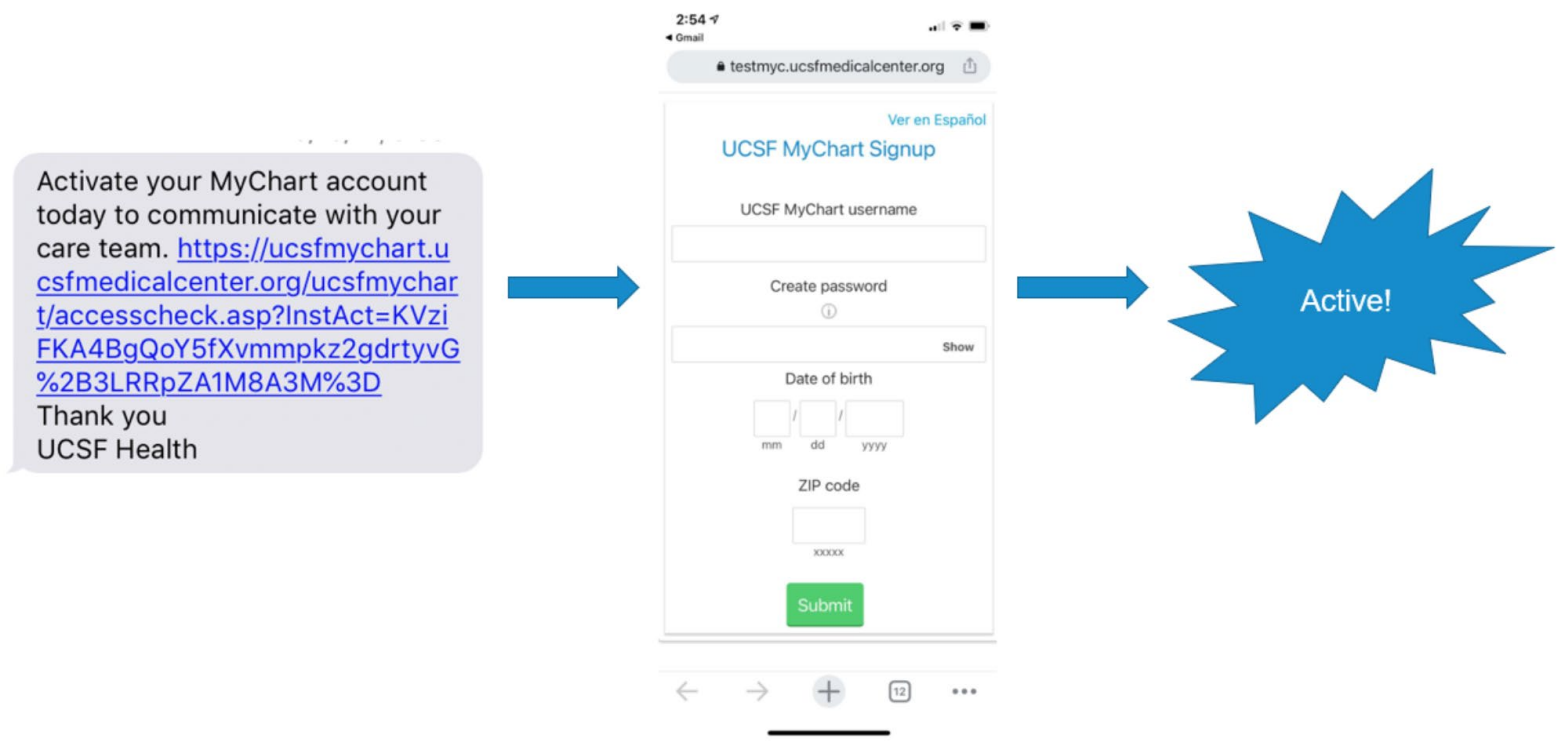
Example of autoenrollment offer text message (left) generating link to complete enrollment in the patient portal (middle) leading to an activated patient portal account (right)

Patients who checked-in or scheduled an outpatient visit which generated an outpatient encounter received a single-click patient portal autoenrollment link to their mobile phone number encouraging sign-up to the patient portal. This system did not require patients to identify patient portal access codes in their after-visit paperwork or depend on staff training and resourcing to provision access codes. Patients could click the link from their phone, confirm their date of birth and zip code, create a username and password, and then start using the patient portal immediately. Patients that did not click the link to enroll were not enrolled in the patient portal (Fig. 1).

### Data Collection

For every outpatient encounter at UCSF between 9/1/2019 and 12/31/2020, patient age, race/ethnicity, primary language, and patient portal activation status data were extracted from our electronic medical record, Epic Systems (Veronia, WI), via the Epic Clarity Database. We leveraged our institution’s algorithmic categorization scheme, developed by our Health Equity Council, to provide standard methodology for race/ethnicity description using the separate data fields of race, ethnicity, and ethnic group documented in the electronic medical record.

### Statistical Analysis

Descriptive statistics were calculated across all encounters, including age, race and ethnicity (White, Black, Latinx, Asian, and Other), and preferred language (English or Other). Mean age and days from the start of the study period were also queried. Mann-Whitney or chi-square testing were used, as appropriate.

An interrupted time series model was fit using individual encounter-level binary outcome data (activation versus no activation) to generate the predicted probability of successful patient portal activation at any time point across the study period. The model was graphically represented to assess the impact of the autoenrollment intervention by comparing three time periods, represented by three corresponding trend lines. The first trend line represents the secular trend of the predicted probabilities of portal activation prior to the intervention (all encounters between 9/1/2019 and 3/17/2020). The horizontal line represents the time of intervention (3/17/2020) and demonstrates the immediate change in predicted probabilities of activation following autoenrollment initiation. The final trend line represents the sustained change in slope of predicted probabilities of activation in the months following the intervention (3/17/2020- 12/30/2020).

All aspects of the model — pre-intervention secular trend, immediate intervention change, and post-intervention sustained change in slope — were allowed to differ by race/ethnicity and primary language populations via inclusion of interaction terms in the model. The intervention impact was determined to be best measured by a trend line representing the slope of mean predicted probabilities in activations over time for all five race/ethnicity categories and both language categories. Therefore, we fit an additive risk model by specifying a generalized linear model with an identity link and a binary outcome (activation or no activation). White was set as the reference race/ethnicity and English as the reference for primary language spoken. Finally, the slopes of the mean predicted probabilities pre- and post-intervention were compared using simple ratios to demonstrate magnitude of change. A similar analytic approach was applied to the pediatric population (ages 17 or less) in which this intervention was not employed.

All analyses for this paper were generated using SAS software, Version 9.4 of the SAS System. Copyright © 2013 SAS Institute Inc. SAS and all other SAS Institute Inc. product or service names are registered trademarks or trademarks of SAS Institute Inc., Cary, NC, USA.

This study was conducted in accordance with ethical guidelines and approved by the UCSF IRB (IRB #21-35405). This study was not a clinical trial and was not determined to require individual participant consent.

## Results

### Demographic Characteristics

In total, 1,048,352 patient encounters were included for assessment of patient portal activation between 9/1/2019 and 12/30/2020 at UCSF. In total, there was a statistically significant increase in patient portal activation from 66.2 to 73.7%. Most of the study encounters were White (pre-intervention: 51.4%; post-intervention: 47.7%) with Asian (17.8%; 16.1%) and Latinx (14.1%; 17.0%) being the next most represented racial groups, though there were statistically significant differences in the racial makeup of the cohort pre versus post intervention. The study encounters were also primarily English (91.6%; 90.7%) speaking (Table 1).

**Table 1:** Demographic characteristics for the study population seen at the university of california, san francisco (UCSF) from 9/1/2019 through 12/30/2020 pre- and post-autoenrollment intervention

	Pre-intervention	Post-intervention	*P*-value
**Age (mean ± SD)**	48.1 ± 24.1	43.3 ± 25.3	< 0.0001
**Days since start of study period (mean ± SD)**	100 ± 57.5	352 ± 80.1	< 0.0001
**Patient portal activation**			< 0.0001
Not activated	139,361 (33.8%)	167,041 (26.3%)	
Activated	272,931 (66.2%)	469,019 (73.7%)	
**Age**			< 0.0001
<18 years	62,278 (15.1%)	145,486 (22.9%)	
≥18 years	350,014 (84.9%)	490,574 (77.1%)	
**Race and ethnicity**			< 0.0001
White	211,712 (51.4%)	303,303 (47.7%)	
Black	24,023 (5.8%)	46,614 (7.3%)	
Latinx	58,272 (14.1%)	108,370 (17.0%)	
Asian	73,262 (17.8%)	102,644 (16.1%)	
Other	45,023 (10.9%)	75,129 (11.9%)	
**Preferred Language**			< 0.0001
English	377,628 (91.6%)	576,919 (90.7%)	
Other	34,664 (8.4%)	59,141 (9.3%)	

### Immediate Change in Patient Portal Activation by Race and Ethnicity and Preferred Language among Adult Patients Post-Autoenrollment

**Fig. 2:**
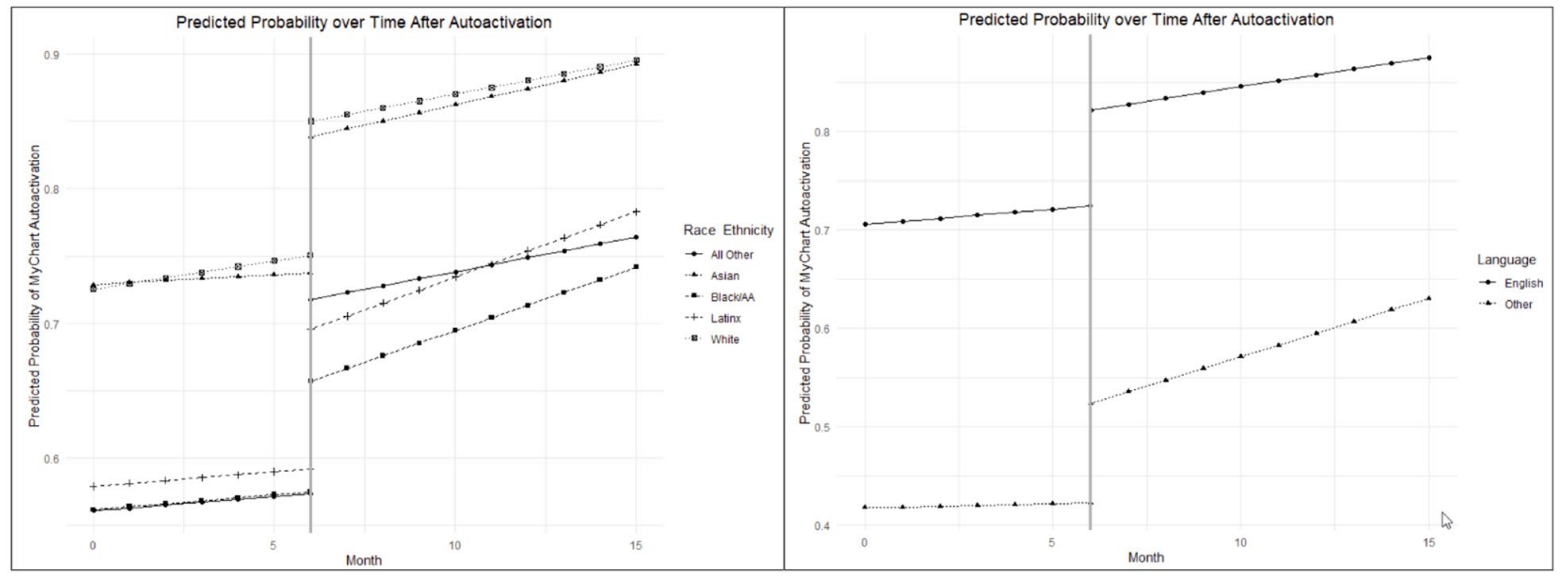
Interrupted time series analysis plotting trend lines representing predicted patient portal (MyChart) activation pre- vs. post-autoenrollment (onset noted by solid vertical line) by race and ethnicity (top) and primary language (bottom) among adult patients (≥ 18 years) seen at the University of California, San Francisco (UCSF) between 9/1/2019 and 12/30/2020. The changes in slope post-intervention represent the sustained change in patient portal activation by racial and/or ethnic group (top) or primary language (bottom) over time

All patients in the groups offered autoenrollment saw a similar immediate increase in enrollment in the patient portal system (Table 2 - Initial Change; Fig. 2, left). These increases were greatest in the Latinx (17.5%, *p* = 0.53), Black (14.3%, *p* = 0.06), and Asian (13.7%, *p* = 0.79) groups but were not statistically significant when compared to White subjects (13.3%). The Other race/ethnicity group did see a statistically significant increase (25.2%, p = < 0.001) when compared to White patients. Similarly, increases in the predicted probability were seen in patients whose preferred language was not English (23.9%) compared with English preferred language patients (13.4%) immediately after the change, although this was not statistically significant (*p* = 0.71).

### Change over Time in the Predicted Probability of Patient Portal Activation by Race and Ethnicity and Preferred Language among Adult Patients Post-Autoenrollment

Following this intervention over time (Table 2 - Change over Time; Fig. 2, right), there were statistically significant increases in activation for Latinx (3.5-fold, p = < 0.001), Black (3.2-fold, *p* = 0.003), and Asian (3.1-fold, *p* = 0.002) patients, as compared to White patients (1.2-fold). Changes in the group of Other race and/or ethnicity increased over time (1.5-fold) but were not statistically significant (*p* = 0.3012), as compared to White patients. Increases over time in patients with a preferred language other than English (13-fold) were also statistically significant (p = < 0.001) when compared with the increase in English preferred language (93.2%) patients.

**Table 2:** Interrupted time series analysis of the initial percent change and percent change over time of the trend lines representing predicted probability of patient portal (MyChart) activation by race and ethnicity (compared to White race as the reference group) and primary language (compared to English as the reference group) among adult patients (≥ 18 years) in whom auto-enrollment was offered seen at the University of California, San Francisco (UCSF) between 9/1/2019 and 12/30/2020

	Initial Change		Change Over Time			
	Percentage Point Change (%) at Singular Instance of Intervention	P-value (compared ref group)	Pre-Intervention Predictive Probability Slope	Post-Intervention Predictive Probability Slope	Ratio of Model Pre- and Post- Slopes	P-value (compared ref group)
**Race and Ethnicity**						
White	13.3	*Ref*	0.0042	0.0050	1.2	*Ref*
Black	14.3	0.06	0.0022	0.0094	3.2	0.003
Latinx	17.5	0.53	0.0022	0.0097	3.5	< 0.001
Asian	13.7	0.79	0.0015	0.0060	3.1	0.002
Other	25.2	< 0.0001	0.0021	0.0052	1.5	0.30
**Preferred Language**						
English	13.5	*Ref*	0.0031	0.0060	1.9	*Ref*
Other	23.9	0.71	0.0008	0.0119	13.0	< 0.001

### Immediate Change and Change over Time of Patient Portal Activation by Race and Ethnicity and Preferred Language among Pediatric Proxy Subjects not Offered Autoenrollment

Pediatric proxy subjects not offered autoenrollment during the same period (Supplemental Table 1 and Supplemental Fig. 1) were separately analyzed. These groups showed no significant differences in Latinx (10.6%, *p* = 0.40), Black (12.5%, *p* = 0.87), Asian (7.2%, *p* = 0.94), Other race and ethnicity (14.2%, *p* = 0.17) as compared to White patients (8.09%) with the initial change after autoenrollment. There were also no statistically significant differences over time in activation in Latinx (-2.1-fold, *p* = 0.17), Black (2.7-fold, *p* = 0.26), Asian (3-fold, 0.38), or Other race and ethnicity (1.6-fold, *p* = 0.90), as compared to White patients (1.6-fold). Pediatric proxy subjects whose preferred language was not English did have an increase in the immediate predicted probability of patient portal activation that approximated statistical significance (16.5%, *p* = 0.06) as compared to English preferred language patients (8.16%). However, they had a 0.3-fold decrease in predicted probability of activation over time as compared with English preferred language patients whose predicted probability rose 2-fold, also approximating statistical significance (*p* = 0.06).

## Discussion

Patient portals improve outcomes across various diseases, yet disparities in access persist between racial, ethnic, and language-based groups, posing a threat of worsening health outcome inequity [6–35, 47–53]. Building on past interventions, we implemented an automated patient portal enrollment text-message workflow [27, 63, 70]. In our cohort of adult patients at UCSF, autoenrollment increased activation across all groups and decreased racial, ethnic, and language-related disparities in patient portal activation over time. Our results suggest automatic patient portal enrollment, a feasible and scalable intervention, may be helpful in mitigating disparities when adopted into the workflow of healthcare organizations.

Portal activation increased immediately across all groups after implementing autoenrollment. However, the magnitude of activation varied between demographic groups over the following nine months. Initially, Latinx, Black, and Asian patients experienced larger activation increases than White patients, though these differences were not statistically significant. Patients whose preferred language was not English also saw greater activation increases than English-speaking patients, but this was not statistically significant at the start. Over nine months, differences between groups became statistically significant. Latinx, Black, and Asian patients, who had lower pre-intervention enrollment rates, saw significantly higher activation increases than White patients. The same trend was observed for non-English-speaking patients. While raw enrollment disparities persisted, our results suggest that autoenrollment offers may reduce activation rate disparities.

Although devised before the COVID-19 pandemic, our intervention coincided with the onset of the pandemic. Multiple studies during and after the pandemic have shown continued disparities in patient portal access and use between minoritized groups and White, English-speaking patients [19, 21, 22, 71–82]. Notably, our study demonstrated that while overall activation increased and pre-pandemic disparities in raw enrollment numbers persisted, the magnitude of these disparities decreased significantly over nine months of follow-up. To understand if the effects on racial and language-based patient portal disparities were related to increased HIT use overall due to the onset of the pandemic rather than the intervention itself, we collected data on the trends of the pediatric proxy population who did not receive autoenrollment during the intervention period. While this does not serve as a true control group for direct comparison, it is notable that the pediatric proxy population did not demonstrate similar trends in improvement in patient portal enrollment among minoritized groups and non-English speakers. In fact, among non-primary English speakers, patient portal enrollment decreased over time, worsening the pre-existing disparity. Similarly, racial, and ethnic disparities also persisted or widened in the pediatric proxy group – specifically, the Latinx population saw a decrease in enrollment rates over time, also worsening the pre-existing disparity. This suggests that the narrowing of disparities among adult subjects may be attributed to the intervention itself. Implementing a similar intervention to a pediatric proxy population will be an important next step given unique modifying factors including proxy access. Nonetheless, the generalizability of this study in a post-pandemic world must be replicated and validated.

The distinction between patient portal enrollment versus patient portal use is a limitation of this study, as we did not track or analyze patterns of use, such as active sessions, repeat logins, or functionality tallying [62, 83]. Prior work has demonstrated that improving patient portal activation does correspond to increased patient portal use, though other studies show conflicting trends [21, 26, 33, 35, 84, 85]. A recent study by Mai et al., which examined patient portal use during the pandemic, found that while the digital divide in portal use persisted, its extent decreased – although marginalized patients had less access to portals, those that did have access used more portal functions [83]. The reduction identified was associated with minoritized groups preferentially accessing portals via mobile devices [83, 86–91]. This finding complements our study, as our intervention used text messages sent to patients’ mobile devices. We therefore hypothesize that successful autoenrollment via text message could lead to active engagement with the patient portal system for our study population. However, we note that it is essential for future organization-based interventions to investigate post-enrollment portal use.

Other limitations of this study include the lack of a direct control group for comparison as well as the absence of information of other potential individual, technology, and environment factors that could impact successful patient portal enrollment, including insurance status, internet or mobile phone access, or other social determinants of health. Additionally, this study had an abbreviated follow-up period, which limits our ability to assess the sustainability of autoenrollment impact over years. Finally, we again emphasize that given the inadvertent overlap with the COVID pandemic, the generalizability of this study in a post-pandemic world must be replicated and validated.

In conclusion, our organization-based intervention of portal enrollment workflow modification with automated enrollment was associated with statistically significant mitigation of racial, ethnic, and language-based portal activation rate disparities. Although promising, the autoenrollment intervention did not eliminate disparities in portal enrollment. Additionally, by definition, our organization-based intervention did not study the individual-, technology-, and environment-dependent barriers and facilitators to portal enrollment. Further research into the synergistic and antagonistic effects of such variables in the setting of autoenrollment may identify areas for further workflow improvement. For this reason, we echo the call to action of previous researchers — more work must be done to tackle disparities in access to HIT and patient portals from all angles, using individual, technological, environmental, and organizational interventions simultaneously. Modern technology, including health technologies, will continue to advance. It is imperative to ensure equity and fairness in its development by preventing further disparities so that healthcare quality may improve for all.

## Electronic supplementary material

Below is the link to the electronic supplementary material.

Supplementary Material 1

## Data Availability

No datasets were generated or analysed during the current study.
