# Supplementary material for: Automatic Enrollment in Patient Portal Systems Mitigates the Digital Divide in Healthcare: An Interrupted Time Series Analysis of an Autoenrollment Workflow Intervention
Source: J Med Syst. 2024 Oct 8;48(1):94. doi: 10.1007/s10916-024-02114-7 (PMC11461562; doi:10.1007/s10916-024-02114-7)
Supplement: Supplementary file 1 — Supplementary Material 1 [file 10916_2024_2114_MOESM1_ESM.docx]

**Supplemental Table 1:** Interrupted time series analysis of the initial percent change and percent change over time of the predicted probability of patient portal (MyChart) activation by race and ethnicity (compared to White as the reference group) and primary language (compared to English as the reference group) among pediatric patients (≤18 years) in whom auto-enrollment was not offered seen at the University of California, San Francisco (UCSF) between 9/1/2019 and 12/30/2020.

|  | **Initial Change** | | **Change over Time** | | | |
| --- | --- | --- | --- | --- | --- | --- |
|  | Percentage Point Change (%) | P-value  (compared ref group) | Pre-Slope Predictive Probability | Post-Slope  Predictive Probability | Ratio of Model Predictive Probability | P-value  (compared ref group) |
| **Race and Ethnicity** |  |  |  |  |  |  |
| White | 8.1 | *Ref* | 0.0046 | 0.0071 | 1.6 | *Ref* |
| Black | 12.5 | 0.87 | 0.0034 | 0.0125 | 2.7 | 0.26 |
| Latinx | 10.6 | 0.40 | 0.0085 | 0.0067 | -2.1 | 0.17 |
| Asian | 7.2 | 0.94 | 0.0019 | 0.0076 | 3.0 | 0.38 |
| Other | 14.2 | 0.17 | -0.0019 | 0.0012 | 1.6 | 0.90 |
| **Preferred Language** |  |  |  |  |  |  |
| English | 8.2 | *Ref* | 0.0036 | 0.0073 | 2.0 | *Ref* |
| Other | 16.5 | 0.05 | 0.0054 | 0.0037 | -0.3 | 0.06 |

**Supplemental Figure 1:** Interrupted time series analysis plotting trend lines of predicted probability of patient portal (MyChart) activation by race and ethnicity (top) and primary language (bottom) among pediatric patients (≤18 years) in whom auto-enrollment was not offered seen at the University of California, San Francisco (UCSF) between 9/1/2019 and 12/30/2020. The changes in slope post-intervention represent the sustained change in patient portal activation by racial and/or ethnic group (top) or primary language (bottom) over time.

_
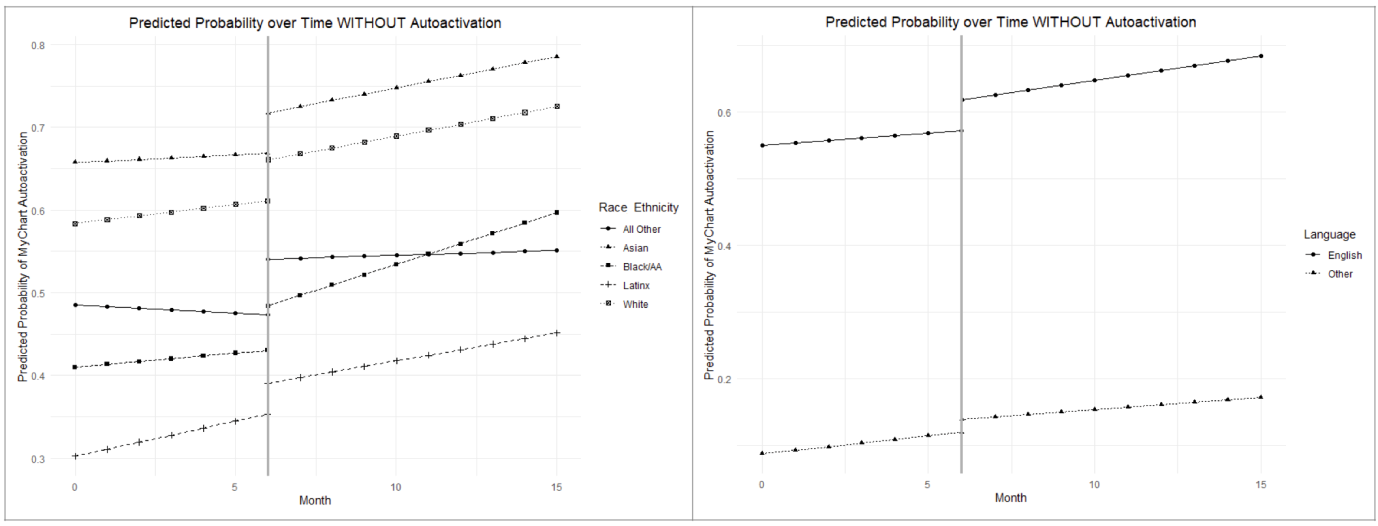
_
